# Novel Cerebello-Amygdala Connections Provide Missing Link Between Cerebellum and Limbic System

**DOI:** 10.3389/fnsys.2022.879634

**Published:** 2022-05-13

**Authors:** Se Jung Jung, Ksenia Vlasov, Alexa F. D’Ambra, Abhijna Parigi, Mihir Baya, Edbertt Paul Frez, Jacqueline Villalobos, Marina Fernandez-Frentzel, Maribel Anguiano, Yoichiro Ideguchi, Evan G. Antzoulatos, Diasynou Fioravante

**Affiliations:** ^1^Center for Neuroscience, University of California, Davis, Davis, CA, United States; ^2^Department of Neurobiology, Physiology and Behavior, University of California, Davis, Davis, CA, United States

**Keywords:** cerebellar nuclei, basolateral amydala, limbic, circuit, electrophysiology, channelrhodopsin, anatomy, mouse

## Abstract

The cerebellum is emerging as a powerful regulator of cognitive and affective processing and memory in both humans and animals and has been implicated in affective disorders. How the cerebellum supports affective function remains poorly understood. The short-latency (just a few milliseconds) functional connections that were identified between the cerebellum and amygdala—a structure crucial for the processing of emotion and valence—more than four decades ago raise the exciting, yet untested, possibility that a cerebellum-amygdala pathway communicates information important for emotion. The major hurdle in rigorously testing this possibility is the lack of knowledge about the anatomy and functional connectivity of this pathway. Our initial anatomical tracing studies in mice excluded the existence of a direct monosynaptic connection between the cerebellum and amygdala. Using transneuronal tracing techniques, we have identified a novel disynaptic circuit between the cerebellar output nuclei and the basolateral amygdala. This circuit recruits the understudied intralaminar thalamus as a node. Using *ex vivo* optophysiology and super-resolution microscopy, we provide the first evidence for the functionality of the pathway, thus offering a missing mechanistic link between the cerebellum and amygdala. This discovery provides a connectivity blueprint between the cerebellum and a key structure of the limbic system. As such, it is the requisite first step toward obtaining new knowledge about cerebellar function in emotion, thus fundamentally advancing understanding of the neurobiology of emotion, which is perturbed in mental and autism spectrum disorders.

## Introduction

The cerebellum is increasingly recognized as a regulator of limbic functions (Strick et al., 2009; Buckner, 2013; Reeber et al., 2013; Strata, 2015; Adamaszek et al., 2017; Schmahmann, 2019; Liang and Carlson, 2020; Hull, 2020). The human cerebellum is activated in response to aversive or threatening cues, upon remembering emotionally charged events, and during social behavior, reward-based decision making, and violation of expectations (Ploghaus et al., 1999; Damasio et al., 2000; Ernst, 2002; Ahs et al., 2009; Moulton et al., 2010, 2014; Guo et al., 2013; Van Overwalle et al., 2014; Guell et al., 2018; Ernst et al., 2019). Consistent with this, deficits in cerebellar function are associated with impaired emotional attention and perception, as seen in depression, anxiety, schizophrenia, and post-traumatic stress disorder (Yin et al., 2011; Roy et al., 2013; Parker et al., 2014; Phillips et al., 2015), as well as cognitive and emotional disturbances collectively known as cerebellar cognitive affective syndrome (Schmahmann and Sherman, 1998). Animal models have recapitulated some of these findings, with selective mutations, damage or inactivation of the rodent cerebellum resulting in altered acquisition or extinction of learned defensive responses, and impaired social and goal-directed behavior, without motor deficits (Supple et al., 1987; Supple and Leaton, 1990; Sebastiani et al., 1992; Bauer et al., 2011; Lorivel et al., 2014; Otsuka et al., 2016; Xiao et al., 2018; Carta et al., 2019; Frontera et al., 2020; Han et al., 2021; Baek et al., 2022; Lawrenson et al., 2022).

The limited understanding of the anatomical and functional circuits that connect the cerebellum to limbic centers has impeded mechanistic insight into the neural underpinnings of cerebellar limbic functions, which have begun to be dissected only recently (Xiao et al., 2018; Carta et al., 2019; Frontera et al., 2020; Kelly et al., 2020; Low et al., 2021). Moreover, a neuroanatomical substrate for the functional connections between the cerebellum and a key affective center, the amygdala (Janak and Tye, 2015), has yet to be provided, even though these connections were observed more than 40 years ago (Heath and Harper, 1974; Snider and Maiti, 1976; Heath et al., 1978). The purpose of the present work was to generate a mesoscale map of functional neuroanatomical connectivity between the cerebellum and amygdala. We focused on connections between the deep cerebellar nuclei (DCN), which give rise to most cerebellar output pathways (Ito, 2006), and the basolateral amygdala (BLA), which is known to process affect-relevant salience and valence information (Janak and Tye, 2015; O’Neill et al., 2018; Yizhar and Klavir, 2018), and which was targeted in the early electrophysiological studies of Heath and Harper (1974) and Heath et al. (1978).

## Materials and Methods

### Mice

C57Bl/6J mice of both sexes were used in accordance with National Institute of Health guidelines. All procedures were reviewed and approved by the Institutional Animal Care and Use Committee of the University of California, Davis. Mice were maintained on a 12-h light/dark cycle with ad libitum access to food and water. For anatomical tracing experiments, postnatal day P45–65 (at the time of injection) mice were used (*N* = 11 mice). For slice optophysiology, P18–25 (at the time of injection) mice were used.

### Virus and Tracer Injections

For stereotaxic surgeries, mice were induced to a surgical plane of anesthesia with 5% isoflurane and maintained at 1%–2% isoflurane. Mice were placed in a stereotaxic frame (David Kopf Instruments, Tujunga, CA) on a feedback-controlled heating pad. Following the skin incision, small craniotomies were made above the target regions with a dental drill. The following coordinates (in mm) were used (from bregma): for medial DCN: −6.4 AP, ± 0.75 ML, −2.2 DV; for interposed DCN: −6.3 AP, ± 1.6 ML, −2.2 DV; for lateral DCN: −5.7 AP, ± 2.35 ML, −2.18 DV. For basolateral amygdala: −0.85 AP, ± 3.08 ML, −4.5 DV. For limbic thalamus: −0.85 AP, ± 0.3 ML, −3.3 DV, and −1.2 AP, ± 0.5 ML, −3.5 DV. A small amount of tracer (50–100 nl for DCN, 300–500 nl for thalamus) was pressure-injected in the targeted site with a UMP3–1 ultramicropump (WPI, Sarasota, FL) and glass pipettes (Wiretrol II, Drummond; tip diameter: 25–50 μm) at a rate of 30 nl/min. The pipette was retracted 10 min after injection, the skin was sutured (Ethilon P-6 sutures, Ethicon, Raritan, NJ) and/or glued (Gluture, Abbott Labs, Abbott Park, IL) and the animal was allowed to recover completely prior to returning to the home cage. Preoperative analgesia consisted of a single administration of local lidocaine (VetOne, MWI, Boise, ID; 1 mg/kg) and Meloxicam (Covetrus, Portland, ME; 5 mg/kg), both SC. Postoperative analgesia consisted of a single administration of Buprenex (AmerisourceBergen Drug Corp, Sacramento, CA; 0.1 mg/kg) and Meloxicam 5 mg/kg, both SC, followed by Meloxicam at 24 and 48 h. The following adeno-associated viruses (AAV) and tracers were used: AAV8-CMV-TurboRFP (UPenn Vector Core, 1.19*10^14^ gc/ml), AAV9-CAG-GFP (UNC Vector Core, 2 × 10^12^ gc/ml), AAV2-retro-CAG-GFP (Addgene, 7 × 10^12^ gc/ml), AAV2-retro-AAV-CAG-tdTomato (Addgene, 7 × 10^12^ gc/ml), Cholera toxin subunit B CF-640 (Biotium, 2 mg/ml, 100 nl), AAV1-hSyn-Cre-WPRE-hGH (Addgene, 10^13^ gc/ml, diluted 1:5), AAV5-CAG-FLEX-tdtomato (UNC Viral Core, 7.8*10^12^ gc/ml, diluted 1:5), AAV9-EF1a-DIO-hChR2(H134R)-EYFP (Addgene, 1.8*10^13^ gc/ml, diluted 1:10), AAV2-hSyn-hChR2(H134R)-EYFP (UNC Vector Core, 5.6 × 10^12^ gc/ml, diluted 1:2). Three to 5 weeks were allowed for viral expression/labeling.

### Histology and Imaging

Following deep anesthesia (anesthetic cocktail: 100 mg/kg ketamine, 10 mg/kg xylazine, 1 mg/kg acepromazine, IP) mice were paraformaldehyde-fixed (4% paraformaldehyde in 0.1 M phosphate buffer, pH 7.4, EMS Diasum, Hatfield, PA) through transcardial perfusion. Brains were post-fixed overnight, cryo-protected with 30% sucrose in PBS, and sliced coronally on a sliding microtome at 60–100 μm thickness. Slices were mounted on slides with Mowiol-based mounting media and scanned using an Olympus VS120 Slide Scanner (Olympus, Germany; resolution with 10× 0.4 N.A. lens at 488 nm: 645 nm in x, y). For immunohistochemistry, slices were blocked with 10% normal goat serum (NGS, Millipore, Burlington, MA) in PBST (0.3% Triton X-100 in PBS) for 1 h. Slices were incubated with primary antibodies (anti-Cre IgG1, Millipore, 1:1,000; anti-NEUN, Cell Signaling, Danvers, MA, 1:1,000; anti-vGLUT2, Synaptic Systems, Goettingen, Germany, 1:700; anti-PSD-95, Neuromab, Davis, CA, 1:500) in 2% NGS-PBST overnight at 4°C. After 4× 20-min rinses with PBST, secondary antibodies (Alexa fluor-568 goat anti-mouse 1:1,000 IgG1; Alexa fluor-488 goat anti-rabbit 1:1,000; Dylight-405 goat anti-guinea pig 1:200; Alexa fluor-647 goat anti-mouse 1:1,000 IgG2a; Life Technologies, Carlsbad, CA) were applied in 2% NGS-PBST for 1–2 h at room temperature. Following another round of rinses, slices were mounted on slides with Mowiol and scanned on an LSM800 confocal microscope with Airyscan (resolution with 63× 1.4 N.A. oil lens at 488 nm: 120 nm in x, y, 350 nm in z; Zeiss, Germany). Maximal projections of optical *z*-stacks were obtained with Zen software (Zeiss) or ImageJ and used for analysis.

### Preparation of Brain Slices for Electrophysiology

Mice of either sex (P39–60) were anesthetized through intraperitoneal injection of ketamine/xylazine/acepromazine anesthetic cocktail and transcardially perfused with ice-cold artificial cerebrospinal fluid (aCSF; in mM: 127 NaCl, 2.5 KCl, 1.25 NaH_2_PO_4_, 25 NaHCO_3_, 1 MgCl_2_, 2 CaCl_2_, 25 glucose; supplemented with 0.4 sodium ascorbate and 2 sodium pyruvate; ~310 mOsm). Brains were rapidly removed, blocked, and placed in choline slurry (110 choline chloride, 25 NaHCO_3,_ 25 glucose, 2.5 KCl, 1.25 NaH_2_PO_4_, 7 MgCl_2_, 0.5 CaCl_2_, 11.6 sodium ascorbate, 3.1 sodium pyruvate; ~310 mOsm). Coronal sections (250 μm) containing the thalamus were cut on a vibratome (Leica VT1200S) and allowed to recover in aCSF at 32°C for 25 min before moving to room temperature until further use. All solutions were bubbled with 95% O_2_–5% CO_2_ continuously. Chemicals were from Sigma.

### Electrophysiology

Slices were mounted onto poly-l-lysine-coated glass coverslips and placed in a submersion recording chamber perfused with aCSF (2–3 ml/min) at near-physiological temperature (30°C–32°C). Whole-cell voltage-clamp recordings were made from tdTomato+ (Figures 3, 5) or CtB+ (Figure 6) cells in the thalamus using borosilicate glass pipettes (3–5 MΩ) filled with internal solution containing (in mM): CsMSO_3_ 120, CsCl 15, NaCl 8, TEA-Cl 10, HEPES 10, EGTA 0.5, QX314 2, MgATP 4 and NaGTP 0.3, biocytin 0.3. Recordings were acquired in pClamp11 using a Multiclamp 700B amplifier (Molecular Devices, San Jose, CA), digitized at 20 kHz, and low-pass filtered at 8 kHz. Membrane potential was maintained at −70 mV. Series resistance and leak current were monitored and recordings were terminated if either of these parameters changed by more than 50%. Optical stimulation of ChR2+ fibers surrounding tdTomato+ or CtB+ thalamic neurons was performed under a 60x water immersion lens (1.0 N.A.) of an Olympus BX51W microscope, using an LED system (Excelitas X-cite; or Prizmatix UHP-T) mounted on the microscope and driven by a Master9 stimulator (AMPI). Optical stimulation consisted of 488 nm light pulses (1–5 ms duration). Power density was set to 1.5–2× threshold (max: 0.25 mW/mm^2^). A minimum of five response-evoking trials (inter-trial interval: 60 s) were delivered and traces were averaged. To confirm monosynaptic inputs, action potentials were blocked with TTX (1 μM), followed by TTX+ 4AP (100 μM) to prolong ChR2-evoked depolarization. A connection is monosynaptic if prolonged ChR2-induced presynaptic depolarization in TTX+4AP is sufficient to evoke release (Petreanu et al., 2009).

**Figure 1:**
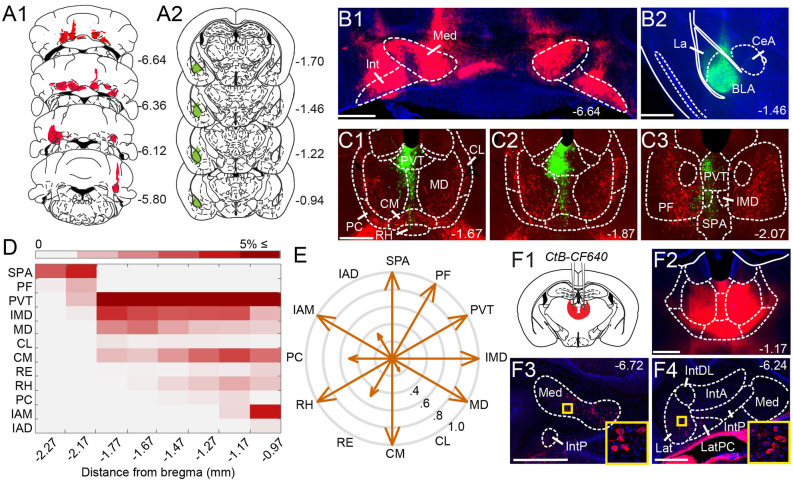
Anatomical tracing uncovers putative disynaptic pathways from the cerebellum tobasolateral amygdala. **(A)** Injection sites for anterogradeviral tracer in DCN (**A1**, *red*) and retrograde viraltracer in BLA (**A2**, *green*). **(B)** Mosaic epifluorescence image of injection sites in DCN **(B1)** and BLA **(B2)**. **(C1–C3)** Mosaic epifluorescence images of overlapping DCN axons (red) and BLA-projecting neurons (green) in limbic thalamus. **(D)** Relative distribution of BLA-projecting neurons across nuclei of the limbic thalamus, normalized to the total number of labeled neurons and averaged across experiments, as a function of distance from bregma. Antero-posterior coordinates for each nucleus are given in Table 1. **(E)** Quantification of overlap between DCN axons and BLA-projecting thalamic neurons. Arrow length in compass plot indicates proportion (0.0–1.0) of experiments with overlap in each thalamic nucleus. **(F1,F2)** Schematic and confocal image of injection site for retrograde tracer CtB CF-640 in limbic thalamus. **(F3,F4)** CtB-labeled projection neurons (red) in DCN at different distances from bregma. Insets show high-magnification images of areas in yellow squares. For all panels, numbers denote distance (in mm) from bregma. Blue: DAPI. Scale bars: 500 μm.

**Figure 2:**
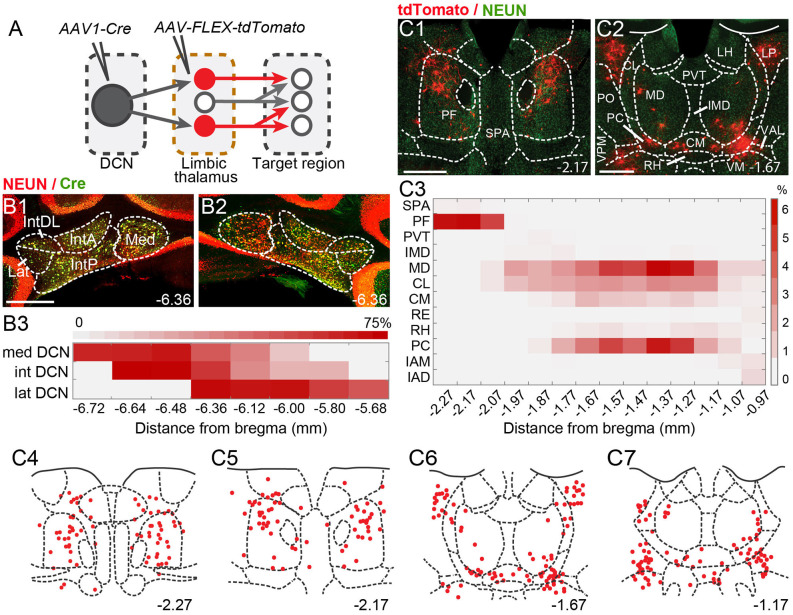
Theintralaminar and mediodorsal nuclei are major cerebellar postsynaptic targets in the limbic thalamus.** (A)** Schematic of experimental approach for disynaptic pathway tracing. **(B1,B2)** Example images of bilateral Cre expression in DCN. Red: immunofluorescence for NeuN neural marker; Green: anti-Cre immunoreactivity; Yellow: merge. **(B3)** Heatmap of Cre immunofluorescence in DCN, normalized to NeuN signal and averaged across experiments, as a function of distance (in mm) from bregma. **(C1,C2)** Example images of thalamic neurons conditionally expressing tdTomato (red) upon transneuronal transfer of Cre from cerebellar presynaptic axons. Green: NeuN immunofluorescence. **(C3)** Heatmap of the relative distribution of tdTomato+ neurons across thalamic nuclei, normalized to the total number of labeled neurons and averaged across experiments, as a function of distance from bregma. **(C4–C7)** Example registration of tdTomato+ neurons to the Allen mouse brain atlas. Numbers at the bottom denote distance (in mm) from bregma. Antero-posterior coordinates for each nucleus can be found in Table 1. Scale bars: 500 μm.

**Figure 3:**
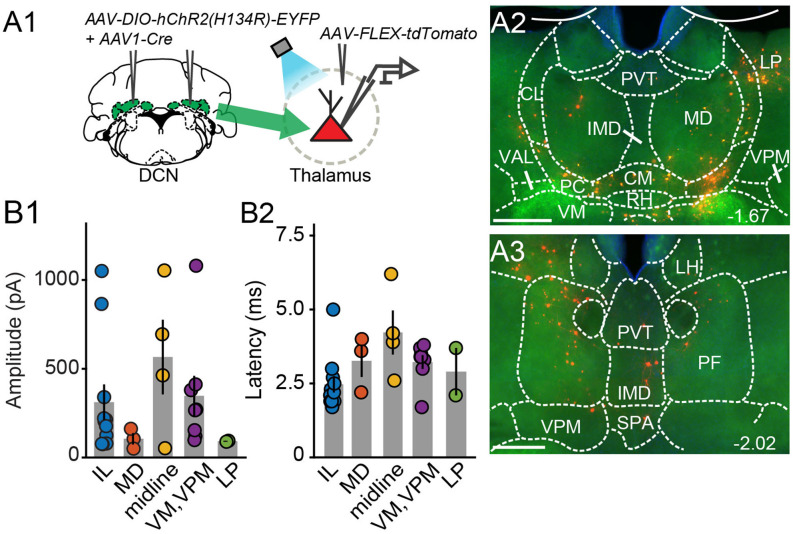
Electrophysiologicalvalidation of virally-identified cerebello-thalamic connectivity. **(A1)** Schematic of experimental approach for *ex vivo* optophysiology. **(A2,A3)** Epifluorescence images of anterior **(A2)** and posterior **(A3)** thalamic slices acutely prepared for recordings. DCN input-receiving neurons are tdTomato+. Scale bars: 500 μm. **(B)** Average (± SEM) amplitude **(B1)** and onset latency **(B2)** of ChR2-evoked synaptic currents as a function of recording location in the thalamus. Intralaminar (IL) group: CL, PC, CM, and PF; midline group: IMD and RH.

**Figure 4:**
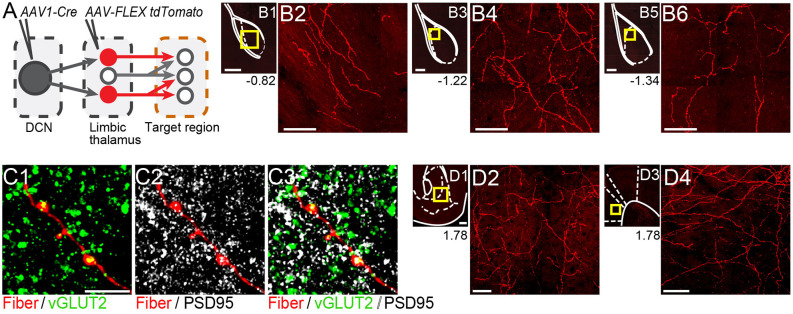
Thalamicneurons receiving cerebellar input form synapses in the basolateral amygdala and also target the nucleus accumbens and prelimbic cortex. **(A)** Schematic diagram of the experimental approach. Targets of tdTomato+ axons of thalamic neurons receiving cerebellar input were identified through imaging. **(B)** Mosaic confocal images of tdTomato+ axons along the anterior-posterior axis of the BLA. **(C)** High resolution airyscan confocal images of tdTomato+ axons in the BLA colocalizing with presynaptic (vGLUT2) **(C1)** and postsynaptic (PSD95) **(C2**) markers of excitatory synapses. Green: vGLUT2, gray: PSD95, yellow/white in **(C3)**: overlay. **(D)** tdTomato+ axons in nucleus accumbens **(D1,D2)** and prelimbic cortex **(D3,D4)**. Yellow squares in **(B1,B3,B5,D1,D3)** show zoom-in areas for **(B2,B4,B6,D2,D4)** images, respectively. Numbers at the bottom of images indicate the distance (in mm) from bregma. Scale bars: **(B1,B3,B5,D1,D3)**: 200 μm; **(B2,B4,B6,D2,D4)**: 50 μm; **(C1–C3)**: 5 μm.

**Figure 5:**
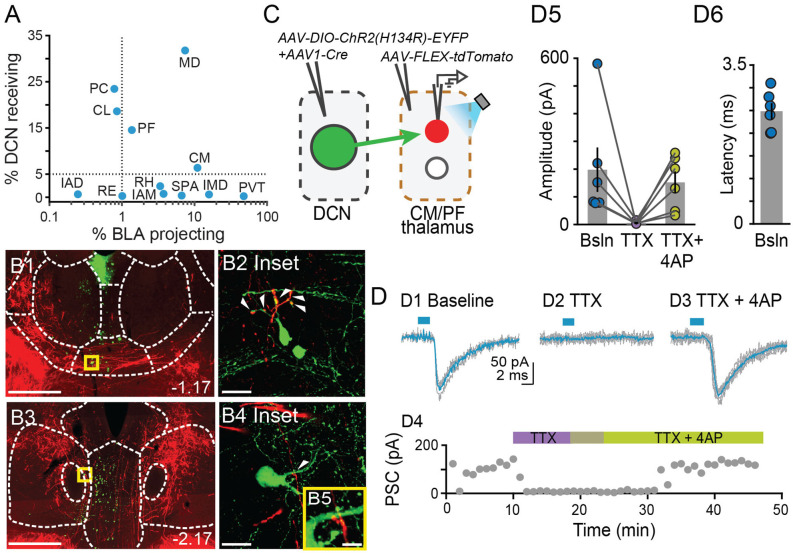
Centromedial and parafascicular neurons project to the basolateral amygdala andreceive functional monosynaptic input from the cerebellum.**(A)** Scatterplot of % neurons receiving DCN input vs. % neurons projecting to BLA, for limbic thalamus nuclei. **(B1–B4)** Airyscan confocal images of DCN axons (red) and BLA-projecting neurons (green) in the centromedial (CM; **B1**) and parafascicular (PF; **B3**) thalamic nuclei. **(B2,B4,B5)** Zoomed-in areas in yellow squares from **(B1,B3)**. Scale bars: **(B1,B3)**: 500 μm; **(B2,B4)**: 20 μm; **(B5)**: 5 μm. **(C)** Schematic diagram of *ex vivo* optophysiology approach to test for monosynaptic connections between DCN and CM/PF thalamic n. **(D1–D3)** Average ChR2-evoked synaptic current (teal), overlaid onto single trial responses (gray), at baseline **(D1)**; upon addition of the action potential blocker tetrodotoxin (TTX, 1 μm; **D2**); after further addition of the potassium channel blocker 4-aminopyridine (4AP, 100 μm; **D3**). **(D4)** Time course of the wash-in experiment for the same example cell. **(D5)** Summary of effects on amplitude (mean ± SEM) of ChR2-evoked synaptic responses for the indicated conditions. Bsln: baseline. **(D6)** Average (± SEM) onset latency of ChR2-evoked responses at baseline.

**Figure 6:**
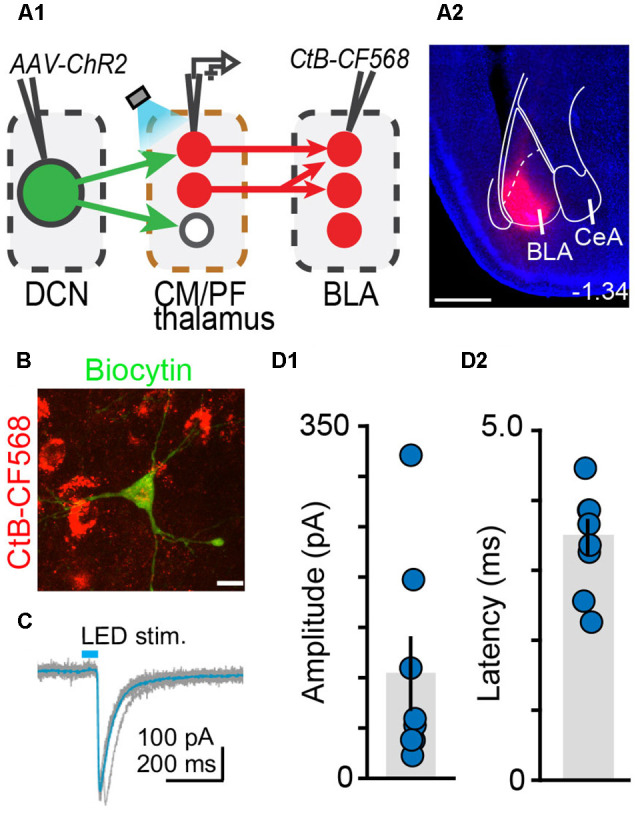
The centromedial and parafascicular thalamus is a functional node of the cerebello-amygdala circuit.** (A)** Experimental approach **(A1)** and example CtB-CF568 injection in amygdala **(A2)**. Blue: DAPI. Scale bar: 500 μm. **(B)** BLA-projecting neuron in centromedial (CM) thalamus retrogradely labeled with CtB CF-568 (red) is also labeled with biocytin (green) through the patch pipette. Scale bar: 10 μm. **(C)** Example ChR2-evoked synaptic response. Average trace (teal) overlaid onto single trials (gray). **(D1,D2)** Average (± SEM) amplitude **(D1)** and onset latency **(D2)** of ChR2-evoked synaptic currents at DCN-CM/PF synapses.

### Data Analysis

Analysis of *ex vivo* recordings was performed using custom MATLAB R2019b scripts (MathWorks, Natick, MA). Postsynaptic current (PSC) amplitude was computed from the maximum negative deflection from baseline within a time window (2.5–40 ms) from stimulus onset. Onset latency was measured at 10% of peak amplitude. Cell location was confirmed through biocytin-streptavidin Alexa fluor staining. For slice registration, the Paxinos Brain Atlas (Paxinos and Franklin, 2001) and the Allen Brain Atlas (ABA_v3) were used. The location of injection sites was identified and experiments were excluded if there was a spill into neighboring nuclei. Cell counting and immunofluorescence intensity analyses were done by raters blind to the experimental hypotheses using ImageJ (Fiji, National Institutes of Health, Bethesda, MD) and Abode Illustrator. Overlap in x- and y-axes between DCN axons and BLA-projecting thalamic neurons was determined through visual inspection of epifluorescence images and evaluated by two independent raters. We note that the resolution of epifluorescence imaging is too low to allow firm conclusions about overlap in the *z*-axis. Statistical analysis was performed in Matlab (Mathworks) and Prism (GraphPad), with significance set at *p* < 0.05. Please see Table 1 for anatomical abbreviations.

**Table 1:** Anatomical abbreviations (in alphabetical order) and antero-posterior coordinates (in mm, from bregma).

Abbreviation	Structure	AP coordinates
BLA	Basolateral amygdaloid nucleus	−0.67 mm to −3.07
CeA	Central amygdala	−0.57 mm to −2.07
CL	Central lateral nucleus of the thalamus	−0.97 mm to −1.97
CM	Central medial nucleus of the thalamus	−0.67 mm to −1.97
DCN	Deep cerebellar nuclei	
IAM	Interanteromedial thalamic nucleus	−0.77 mm to −1.07
IMD	Intermediodorsal nucleus of the thalamus	−0.87 mm to −2.07
Int	Interposed cerebellar nucleus	−6.64 mm to −5.8
IntA	-anterior part	
IntDL	-dorsolateral part	
IntP	-posterior part	
La	Lateral amygdaloid nucleus	−0.87 mm to −2.47
Lat	Lateral cerebellar nucleus	−6.36 mm to −5.68
LP	Lateral posterior thalamic nucleus	−1.27 mm to −3.17
Med	Medial cerebellar nucleus	−6.84 mm to −5.88
MD	Mediodorsal nucleus of the thalamus	−0.57 mm to −1.97
NAc	Nucleus accumbens	
PC	Paracentral nucleus of the thalamus	−1.07 mm to −1.87
PF	Parafascicular nucleus	−1.97 mm to −2.37
PVT	Paraventicular thalamus	−0.17 mm to −2.07
PO	Posterior thalamic nucleus	−1.27 mm to 2.37
PrL	Prelimbic cortex	
RE	Reuniens thalamic nucleus	−0.37 mm to −1.77
RH	Rhomboid thalamic nucleus	−0.77 mm to −1.67
SPA	Subparafascicular area	−2.07 mm to −2.27
VL	Ventrolateral thalamic nucleus	−0.67 mm to −2.27
VM	Ventromedial thalamic nucleus	−0.67 mm to −2.07

## Results

### Putative Disynaptic Pathways Between Cerebellar Nuclei and BLA Through the Limbic Thalamus

Given that microstimulation of DCN elicits short-latency responses in the BLA (Heath and Harper, 1974; Snider and Maiti, 1976; Heath et al., 1978), we hypothesized that an anatomical pathway exists between the two regions that involve at most two synapses. Initial anatomical tracing experiments did not support a direct DCN-BLA connection (not shown). We, therefore, performed simultaneous injections of an anterograde tracer virus (AAV8-CMV-TurboRFP) bilaterally in the DCN and a retrograde tracer virus (AAV2-retro-CAG-GFP) unilaterally in the BLA (Figures 1A,B) to identify potential regions of overlap. In epifluorescence images of brain slices across different animals (*N* = 6), the limbic thalamus consistently emerged as a prominent site of overlap (Figures 1C1–C3). We use the term “limbic thalamus” to refer to a collection of non-sensorimotor thalamic nuclei, including the mediodorsal (MD), midline, and intralaminar (IL) nuclei, with diverse projections to cortical (mainly medial prefrontal) and/or subcortical limbic structures (Groenewegen and Witter, 2004; Morgane et al., 2005; Jones, 2007; Vertes et al., 2015). Registration of images to the Allen Brain Atlas localized BLA-projecting thalamic neurons in multiple nuclei of the limbic thalamus (Figure 1D), in agreement with known connectivity patterns (Van der Werf et al., 2002; Vertes et al., 2015; Amir et al., 2019; Hintiryan et al., 2021). Visual inspection of diffraction-limited epifluorescence images identified overlapping DCN axonal projections and BLA-projecting neurons in several (but not all) of these thalamic nuclei, including the parafascicular (PF) n. and subparafascicular area (SPA), the centromedial (CM) and MD nuclei, and other midline nuclei (Figure 1E). No BLA-projecting neurons were observed in DCN, and no direct DCN projections were observed in BLA (not shown). Injection of the tracer cholera toxin subunit B (CtB)-CF640 in the limbic thalamus retrogradely labeled neurons in all DCN (Figure 1F), confirming the DCN-limbic thalamus connectivity.

### Transneuronal Anatomical Tracing and Optophysiology Establish Synaptic Connectivity Between Cerebellar Nuclei and Limbic Thalamus

To spatially resolve synaptic connectivity between DCN and BLA-projecting thalamic nuclei, we adopted an AAV-based transneuronal approach (Zingg et al., 2017). AAV1-Cre in presynaptic neurons is known to propagate across the synapse and induce expression of a floxed tag in postsynaptic neurons, thus identifying synaptic partners (Figure 2A). We injected AAV1-Cre bilaterally in DCN and AAV-FLEX-tdTomato in the thalamus (*N* = 5) and quantified the relative distribution of tdTomato+ neurons in intralaminar and midline thalamic nuclei. Injection coverage for DCN was indicated by Cre immunofluorescence (Figures 2B1,B2) and included all cerebellar nuclei. Great care was taken to avoid spill to extracerebellar areas, which resulted in denser coverage of caudal DCN (Figure 2B3). TdTomato+ neurons were observed throughout the limbic thalamus, confirming adequate coverage, and extended into ventromedial nuclei (Figure 2C), which served as positive control (Gornati et al., 2018; Habas et al., 2019). Averaging the relative distribution of tdTomato+ neurons across five successful experiments revealed that the intralaminar cluster, comprised of centrolateral (CL), paracentral (PC), CM, and PF nuclei (Van der Werf et al., 2002), and MD nucleus encompassed most (~95%) tagged neurons (Figure 2C3), suggesting that these nuclei reliably receive most cerebellar inputs to limbic thalamus. The paraventricular (PVT) nucleus, even though it projects heavily to BLA (Figure 1C) and features overlap between DCN axons and BLA-projecting neurons (Figure 1E), did not appear to receive robust direct DCN input (Figure 2C3).

To confirm that thalamic targets identified with the transneuronal Cre method receive cerebellar synaptic input, we performed optophysiological experiments in acute thalamic slices from mice injected with AAV1-Cre in the DCN and AAV-FLEX-tdTomato in the thalamus (*N* = 14; Figure 3A). To activate cerebellar inputs, channelrhodopsin (ChR2-H134R) was conditionally expressed in DCN through AAV-DIO-ChR2-EYFP injection. DCN axonal projections were stimulated in the thalamus with 488-nm light pulses applied through the objective. Light-evoked synaptic responses were monitored in whole-cell voltage-clamp recordings (*V_m_* = −70 mV) from thalamic neurons, which were selected based on tdTomato expression, their anatomical location, and position in the slice, i.e., surrounded by ChR2-EYFP-expressing axons. In all thalamic nuclei examined (*n* = 29 cells), light stimulation elicited synaptic responses (mean response in pA: IL: 311.7 ± 100; MD: 105.7 ± 32.3; midline: 565.8 ± 209.8; VM/VPM: 347.5 ± 112.3; LP: 91.8 ± 2.7; Figure 3B1) with short latencies (mean latency in ms: IL: 2.5 ± 0.28; MD: 3.3 ± 0.6; midline: 4.2 ± 0.7; VM/VPM: 3.2 ± 0.2; LP: 2.9 ± 0.8; Figure 3B2). These data support the specificity of the anatomical connectivity and establish the existence of active DCN terminals (as opposed to just passing axons) across the limbic thalamus.

### Thalamic Neurons Receiving Cerebellar Input Project to BLA

If the thalamus is a functional node of the disynaptic DCN-BLA circuit, then we would expect to find axons of DCN input-receiving thalamic neurons in BLA. To this end, we imaged BLA-containing slices from transsynaptic Cre experiments (*N* = 5; Figure 4A). We detected tdTomato+ axons at several antero-posterior distances from bregma (Figures 4B1–B6). Using immunohistochemistry with antibodies against pre- and postsynaptic markers of excitatory synapses (vesicular glutamate transporter, vGLUT2; postsynaptic density protein-95, PSD-95), and super-resolution airyscan confocal imaging, we found tight colocalization between tdTomato+ axonal varicosities, vGLUT2 and PSD-95, an example of which is shown in Figure 4C. This finding suggests that axons of thalamic neurons receiving cerebellar input form morphological synapses in the BLA. Axonal projections of DCN input-receiving thalamic neurons were also observed in other limbic regions including the nucleus accumbens core and shell (Figures 4D1,D2) and anterior cingulate/prelimbic cortex (Figures 4D3,D4).

### The Centromedial and Parafascicular Nuclei Emerge as Functional Nodes in Cerebello-Amygdala Circuit

Our tracer overlap studies pointed to multiple thalamic nuclei as potential relays of cerebellar signals to BLA (Figure 1E). Among them, the MD, CM, and PF nuclei showed a higher relative distribution of both BLA-projecting neurons and neurons that receive DCN input (Figures 1D, 2C, 5A). Further inspection of MD images revealed that, despite clear instances of overlap across experiments, DCN input-receiving neurons localized mostly laterally in MD, and BLA-projecting neurons localized mostly medially. Therefore, to maximize chances of success, for the remainder of this study we focused on CM and PF nuclei and sought to substantiate their role as anatomical and functional relays of DCN-BLA connectivity through super-resolution microscopy and optophysiology.

Airyscan confocal imaging of slices from dual-tracer experiments (Figure 1) revealed fluorescently labeled DCN axons (red) in contact with neurons that were retrogradely labeled from the BLA (green) in both CM (Figures 5B1,B2) and PF (Figures 5B3–B5) nuclei. The existence of functional monosynaptic DCN-CM/PF connections was tested in the subset of electrophysiological experiments from Figure 3 that targeted CM/PF neurons (Figure 5C). Under basal conditions, CM/PF neurons received synaptic inputs from the DCN (at *Vm* = −70 mV; average amplitude ± SEM: −197.5 pA ± −80.14, *n* = 6; Figures 5D1,D5) with short onset latency (average latency ± SEM: 2.4 ms ± 0.18; Figure 5D6), which is consistent with direct monosynaptic connections. Application of the sodium channel blocker tetrodotoxin (TTX) abolished the inputs (average amplitude ± SEM: −5.1 pA ± −2.03; Figures 5D2,D4,D5), which recovered upon addition of the potassium channel blocker 4-AP (average amplitude ± SEM: −151.8 pA ± −39.52; Figures 5D3–D5; Friedman’s non-parametric repeated measures ANOVA: *x^2^_r_* = 9, *n* = 6, *p* = 0.008; Dunn’s multiple comparison test: Baseline vs. TTX: *p* = 0.02, Baseline vs. TTX+4AP: *p* = 0.99, TTX vs. TTX+4AP: *p* = 0.01), confirming their monosynaptic nature.

Finally, we tested whether BLA is a target of DCN input-receiving CM/PF neurons (Figure 6). We virally expressed ChR2 in DCN and stimulated cerebellar axonal projections in thalamic slices while recording from BLA-projecting CM/PF neurons (whole-cell voltage clamp mode, *V_m_* = −70 mV), which were retrogradely labeled with CtB-CF568 in BLA (Figures 6A,B). Optogenetic stimulation elicited reliable DCN-CM/PF synaptic responses (average amplitude ± SEM: −104.1 pA ± −37.1, *n* = 8; Figures 6C,D1) with short latency (3.35 ms ± 0.25; Figure 6D2). Combined with the imaging findings (Figure 5), our electrophysiological results argue strongly for a DCN-BLA disynaptic circuit that recruits CM/PF nuclei as a node.

## Discussion

Cerebellar connections with the amygdala have been posited previously but the neuroanatomical substrate of this connectivity has been elusive (Strick et al., 2009; D’Angelo and Casali, 2013; Adamaszek et al., 2017). Here, we obtained insight into cerebello-amygdala circuitry by combining various tracing approaches with advanced imaging and optophysiology. We established the existence of a disynaptic circuit between cerebellar nuclei and BLA, thus providing the first blueprint of cerebello-amygdala connectivity at the mesoscale level. The circuit recruits at least the centromedial and parafascicular thalamic nuclei (Figures 5, 6), and most likely also other nuclei of the limbic thalamus (Figure 1), as relay nodes. In addition, we identified the intralaminar thalamic cluster and MD nucleus as recipients of the majority of cerebellar inputs to the limbic thalamus (Figure 2). Finally, and in addition to BLA, we identified axonal projections of DCN input-receiving thalamic neurons in limbic regions such as nucleus accumbens core and shell and anterior cingulate/prelimbic cortex (Figure 4).

### The Limbic Thalamus as a Target of Cerebellar Inputs

We targeted the limbic thalamus as a conduit of cerebello-amygdala communication because several of its nuclei foster BLA-projecting neurons in close proximity to DCN axons (Figure 1). DCN projections to limbic thalamus have been observed before (Hendry et al., 1979; Haroian et al., 1981; Ichinohe et al., 2000; Fujita et al., 2020; Judd et al., 2021) but the existence of functional synaptic terminals has only been validated for centrolateral and PF intralaminar nuclei (Gornati et al., 2018; Xiao et al., 2018), and never on amygdala-projecting neurons. Our optophysiological experiments also provided the first evidence for the presence of active synaptic connections (as opposed to just passing axons) in paracentral and centromedial (part of intralaminar group), intermediodorsal and rhomboid (part of midline group), and mediodorsal nuclei (Figure 3), expanding the repertoire of non-motor cerebellar targets and paving the way for causal manipulations.

### Technical Considerations

To chart cerebello-amygdala neuroanatomical connections, we used powerful circuit mapping tools including anterograde and retrograde tracer viruses and the transneuronal AAV1-Cre approach (Tervo et al., 2016; Zingg et al., 2017, 2020; Nectow and Nestler, 2020). A distinct advantage of our approach, which combined AAV1-Cre with viral injections of conditionally expressed fluorescent tracers (as opposed to reporter mouse lines), is the ability to definitively point to the thalamus as the source of the observed axonal projections in BLA, nucleus accumbens, and prelimbic cortex—as opposed to e.g., the VTA, which also receives DCN inputs and projects to these regions (Phillipson, 1979; Kuroda et al., 1996; Beier et al., 2015; Breton et al., 2019; D’Ambra et al., 2020). Thus, our approach enabled a conclusive interpretation of anatomical connectivity results. On the other hand, injection coverage/spill and viral tropism (Nectow and Nestler, 2020) need to be considered. Tropism, in particular, could skew the interpretation of disynaptic inputs, as some cell groups in the limbic thalamus might be more efficiently infected by AAVs. Tropism could also explain why recent efforts to trace di- and tri-synaptic cerebellar efferent pathways with herpes simplex viruses did not identify the CM/PF pathway to BLA (Pisano et al., 2021). Lastly, one potential concern could be the propensity of AAVs to be transported in the retrograde direction at high titers (Rothermel et al., 2013; Zingg et al., 2017). To remediate these concerns, we used strict inclusion criteria for injection sites; employed a combination of viral and non-viral anterograde and retrograde tracers; optimized viral titers to minimize retrograde transport; and confirmed circuit connections with slice optophysiology.

### Proposed Functions of the DCN-BLA Circuit

Our discovery of the DCN-BLA connection through the CM/PF thalamic nuclei provides an essential map for future investigation of circuit function. The circuit, which could account for the previously observed short-latency cerebello-amygdala responses (Heath and Harper, 1974), could convey cerebellar information about prediction, salience, and/or valence to BLA, shaped by the intrinsic, synaptic, and integrative properties of the nodes. Indeed, the cerebellum is known to encode such information (Ohmae and Medina, 2015; Wagner et al., 2017; Hull, 2020; Ma et al., 2020; Bina et al., 2021; Shuster et al., 2021), which is also seen in BLA (Paton et al., 2006; Adolphs, 2010; Janak and Tye, 2015; Sengupta et al., 2018; Zhang and Li, 2018; Gründemann et al., 2019; Brockett et al., 2021), and which is thought to be used by CM and PF during aversive conditioning, observational learning and reward-seeking behavior (Jeon et al., 2010; Sengupta and McNally, 2014; Vertes et al., 2015; Xiao et al., 2018; Cover and Mathur, 2021; Rizzi et al., 2021).

We have provided morphological evidence for synaptic connections between cerebello-thalamic axons and BLA neurons (Figure 4). The functional properties of these synapses remain to be determined, as do the cellular identities of the BLA targets. These targets likely include at least BLA principal neurons, which are the major recipients of CM input (Ahmed et al., 2021). The patterns of BLA ensemble activity triggered by distinct cerebello-thalamic inputs could serve different aspects of cerebellum-dependent emotional functionality, which includes modulation of anxiety and learned fear (Sacchetti et al., 2007; Duvarci and Pare, 2014; Tovote et al., 2015; Otsuka et al., 2016; Frontera et al., 2020; Rudolph et al., 2020); the processing of facial emotional expressions (Wang et al., 2017; Ferrari et al., 2018); regulation of emotional reactivity (Turner et al., 2007; Machado et al., 2009); and even reward-driven motivated behavior (Murray, 2007; Bauer et al., 2011; Peterson et al., 2012; Carta et al., 2019).

The BLA is not the sole nucleus in the amygdala complex that receives cerebellar signals (Magal and Mintz, 2014). Similarly, it is unlikely that the CM and PF are the only nuclei serving cerebello-amygdala communication (our findings; and Kang et al., 2021). Further studies are warranted to delineate the complete neuroanatomical and functional landscape of cerebello-amygdala connectivity. Our findings constitute the first step toward this goal.

## Data Availability Statement

The raw data supporting the conclusions of this article will be made available by the authors upon request.

## Ethics Statement

The animal study was reviewed and approved by Institutional Animal Care and Use Committee of the University of California, Davis.

## Author Contributions

SJ, KV, and DF designed the study. SJ, KV, AD, AP, and YI performed experiments. SJ, KV, EA, and DF analyzed data. MB, EF, JV, MF-F, and MA assisted with cell counting. SJ, KV, AD, and DF wrote the manuscript with input from authors. All authors contributed to the article and approved the submitted version.

## Conflict of Interest

The authors declare that the research was conducted in the absence of any commercial or financial relationships that could be construed as a potential conflict of interest.

## Publisher’s Note

All claims expressed in this article are solely those of the authors and do not necessarily represent those of their affiliated organizations, or those of the publisher, the editors and the reviewers. Any product that may be evaluated in this article, or claim that may be made by its manufacturer, is not guaranteed or endorsed by the publisher.
